# G3 2021 Reviewer Index

**DOI:** 10.1093/g3journal/jkab384

**Published:** 2021-12-07

**Authors:** Ruth Isaacson

Below is a list of individuals who have reviewed manuscripts for G3: Genes | Genomes | Genetics during the preparation of Volume 11 (2021). The aim of G3: Genes | Genomes | Genetics is to communicate high-quality, well-described, useful research, across all areas of genetics and genomics. To succeed, we must recognize what meets our criteria for publication, making sure the presentation is accurate, unambiguous, and easily readable. For this, we depend heavily upon our reviewers, who perform this indispensable service without reward. The quality of the journal strongly reflects the quality of their efforts. We apologize to any reviewer whose name has been inadvertently omitted, and we are sincerely grateful to all. 

Abbott, Allison

Abdollahi, Rostam

Adak, Alper

Adams, Sally

Adhikari, Pragya

Adrion, Jeffrey

Ahmad, Kami

Akbarzadeh, Arash

Akdemir, Deniz

Albert, Frank

Alexandre, Pamela

Aleza, Pablo

Alioto, Tyler

Alkema, Mark

Allen, Anna

Alsaadi, Ahlam

An, Yong-Qiang

Anderson, Sarah

Angel, Joe

Antony, Jisha

Aparicio, Oscar

Appel, Bruce

Arbeitman, Michelle

Auber, Robert

Ayte, Jose

Bach, Erika

Bader, Gary

Bähler, Jürg

Bahn, Yong-Sun

Baker, Lydia

Baker, Robert

Bakthavachalu, Baskar

Baldassari, Antoine

Baltrus, David

Bangi, Erdem

Barazandeh, Marjan

Bargelloni, Luca

Barreto, Guillermo

Baryshnikova, Anastasia

Basler, Konrad

Basrai, Munira

Bass, Chris

Bassil, Nahla

Bastiaansen, John

Bastos de Oliveira, Francisco

Batterham, Phillip

Battey, C. J.

Baud, Amelie

Baxter, Simon

Bayer, Phillip

Bean, Tim

Beaudry, Felix

Bedwell, David

Belanger, Faith

Bello, Nora

Belsare, Saurabh

Belzile, François

Bennett, Brian

Benoit, Matthias

Berchowitz, Luke

Bergey, Christina

Berman, Judith

Bernal Rubio, Yeni L.

Berner, Daniel

Bernhardsson, Carolina

Bertuch, Alison

Besson, Mathieu

Betran, Esther

Beukeboom, Leo

Beuning, Penny

Bhattacharya, Arjun

Bianchi, Laura

Billmyre, R.

Bird, Kevin

Blackmon, Heath

Blair, Seth

Blanco, Antonio

Blomberg, Anders

Bloom, Joshua

Bo, Zhu

Bochman, Matthew

Bohutínská, Magdalena

Bokore, Firdissa

Boland, Michael

Bono, Jeremy

Booker, Tom

Bostan, Hamed

Bourgeois, Yann

Bouzakri, Karim

Bowerman, Bruce

Braendle, Christian

Brenton, Zachary

Bresolin, Tiago

Brisson, Jennifer

Broman, Karl

Brook, William

Brooks, Elizabeth

Brown-Guedira, Gina

Brown, Patrick

Brown, Susan

Bruno, Vincent

Bryk, Mary

Buell, C. Robin

Burga, Alejandro

Burgerhout, Erik

Burgess, Sean

Burgess, Shawn

Busoms, Silvia

Bustos-Korts, Daniela

Byers, Kelsey

Byrne, Alexandra

Cahoon, A Bruce

Calabrese, J. Mauro

Calvi, Brian

Cameron, Stephen

Campbell, Kieran

Campbell, Malachy

Cantor, Rita

Cantu, Dario

Cao, Xiaolong

Capote, Tiago

Cappa, Eduardo

Card, Daren

Carelli, Francesco

Carlen, Elizabeth

Carter, Gregory

Castandet, Benoît

Castillo, Kathrina

Castoe, Todd

Cervantes, Sandra

Chakraborty, Mahul

Chalasani, Shrek

Chang, Ching-Ho

Chaturbedi, Amaresh

Chatwin, Warren

Chee, Peng

Chen, Charles

Chen, Ching-Yi

Chen, Chunyu

Chen, Ee Sin

Chen, Hung-Hsin

Chen, Lijiao

Chen, Liyu

Chen, Maoshan

Chen, Yuning

Chen, Z.

Chen, Zhen-Xia

Cheng, Hao

Cherra, Salvatore

Childs, Kevin

Chiou, Kenneth

Chirico, William

Choe, Keith

Chougule, Kapeel

Christensen, Kris

Clark, Erik

Coffing, Gabrielle

Coller, Hilary

Collins, Kevin

Comeron, Josep

Connallon, Tim

Cook, David

Cooper, Tom

Coughlan, Jennifer

Cox, Rachel

Crain, Jared

Cripps, Richard

Croll, Daniel

Cromie, Gareth

Crossa, José

Crouch, Jo

Cuevas, Jaime

Cuomo, Christina

Cussiol, Jose

Cutter, Asher

Das, Arunika

Datta, Rhea

Davenport, Kristen

Davison, Angus

Dawson-Scully, Ken

Dawson, Dean

De Jong, Walter

De Luca, Maria

De Vienne, Dominique

Dearden, Peter

Dell'Acqua, Matteo

Denton, Robert

DePace, Angela

Derbyshire, Mark

Derivery, Emmanuel

Diaz Caballero, Julio

Diaz-Papkovich, Alex

Dickman, Dion

Diepenbrock, Christine

Diers, Brian

Dillon, Marcus

Dimitriadi, Maria

Ding, Haidong

Dittmar, Emily

Doe, Chris

Doitsidou, Maria

Dong, Meng-Qiu

Dong, Zhiguo

Dorn, Kevin

Downing, Tim

Doyle, Vinson

Dracatos, Peter

Dreisigacker, Susanne

Du, Li-Lin

Dudley, Aimee

Dumont, Beth

Duncan, Mara

Dunlap, Jay

Dupuis, Julian

Durvasula, Arun

Dutta, Prasun

Eddy, Sean

Edge, Doc

Edger, Patrick

Edwards, Jode

Ehrenreich, Ian

Ehringer, Marissa

Emery, Gregory

Emery, Patrick

Emms, David

Endelman, Jeffrey

Enders, Laramy

Engebrecht, JoAnne

Ercan, Sevinc

Ergon, Åshild

Erickson, James

Ersoz, Elhan

Espariz, Martin

Estelle, Mark

Etges, William

Extavour, Cassandra

Facette, Michelle

Faircloth, Brant

Falque, Matthieu

Fanara, Juan

Fariello, Maria Ines

Farre, Marta

Fazzio, Thomas

Fernandes, Samuel

Fernandez-Gonzalez, Rodrigo

Ferreira de Carvalho, Julie

Ferris, Martin

Findlay, Geoff

Fisher, Heidi

Fisher, Matthew

Flames, Nuria

Flint-Garcia, Sherry

Fonseca, Luiz

Fontaneto, Diego

Forche, Anja

Forman, Oliver

Forrest, Douglas

França, Monica

Francis, Michael

Fraslin, Clemence

Freddolino, Peter

Freitag, Michael

Fu, Rui

Furlong, Eileen

Gage, Joseph

Galewski, Paul

Gallone, Brigida

Gambino, Giorgio

Gao, Guanjun

Gao, Yuzhen

García, L.

Garg, Angad

Garlovsky, Martin

Gartenberg, Marc

Garzon Martinez, Gina

Gasch, Audrey

Gautier, Mathieu

Gavery, Mackenzie

Gazda, Malgorzata

Geddes-McAlister, Jennifer

Geisbrecht, Erika

Gendrel, Anne-Valerie

Gerstein, Aleeza

Ghose, Aurnab

Gianola, Daniel

Gifford, Robert

Gill, Kulvinder

Gladyshev, Eugene

Golden, Andy

Goloborodko, Anton

Gondro, Cedric

Gong, Lei

Gonzalez-Gonzalez, Andrea

Gonzalez, Beatriz

Gray, Vincent

Greenstein, David

Gresham, David

Grewal, Savraj

Grigorenko, Elena

Groen, Simon

Groh, Jeffrey

Grossman, Sharron

Grotewold, Erich

Grover, Corrinne

Guertin, Michael

Guiguen, Yann

Gundappa, Manu Kumar

Gurumurthy, Channabasavaiah

Gutierrez, Alejandro

Guydosh, Nicholas

Gyllenhaal, Ethan

Haag, Eric

Hague, Michael

Haley, Scott

Hall, Sarah

Hallingbäck, Henrik

Hamelin, Richard

Hansen, Dave

Hanson, Sara

Hao, Weilong

Harbison, Susan

Hardwick, J. Marie

Harkess, Alex

Hart, Michael

Harvey, Simon

Hauser, Elizabeth

Havey, Michael

He, Fei

Heath-Heckman, Elizabeth

Heifetz, Yael

Heigwer, Florian

Hendricks, Will

Henry, Isabelle

Hentges, Kathryn

Heraghty, Sam

Herman, Adam

Hey, Jody

Hickner, Paul

Hidaka, Michio

Higgins, N.

Hinze, Lori

Hirsch, Cory

Hodonsky, Chani

Hoffman, Charles

Hoffmann, Ary

Hogan, Deborah

Holzem, Michaela

Hong, Ray

Horb, Marko

Hornyak, Thomas

Horvath, Robert

Horwitz, Benjamin

Hotaling, Scott

Houry, Walid

Houston, Derek

Hovav, Ran

Howard, Reka

Howarth, Catherine

Howe, Kevin

Howe, LeAnn

Hsueh, Yen-Ping

Hu, Guanjing

Hu, Ying

Huang, Jing

Huang, Wen

Huang, Xi

Huang, Yuheng

Hufford, Matthew

Humble, Emily

Hunter, Kent

Hurd, Thomas

Hurley, Jennifer

Husband, Brian

Huyop, Fahrul

Ikeogu, Ugochukwu

Iobbi-Nivol, Chantal

Iorizzo, Massimo

Isik, Fikret

Jaillard, Sylvie

Jaiswal, Pankaj

Jamann, Tiffany

Jarquin, Diego

Jarquin, Diego

Javal, Marion

Jeffries, Daniel

Jensen, Just

Jesuthasan, Suresh

Ji, Jun-Yuan

Jia, Zhenyu

Jiang, Ning

Jiggins, Francis

Johannesson, Hanna

Johansson, Anna

Johnson, Charles

Johnson, Rory

Jones, John

Jones, Kenneth

Jordan, I.

Jordan, Katherine

Jospin, Maelle

Junfang, Lin

Juvik, John

Kagey, Jacob

Kainer, David

Kamakaka, Rohinton

Kantar, Michael

Karch, Francois

Kärkkäinen, Hanni

Kaufman, Thomas

Kause, Antti

Keightley, Peter

Kelly, Tara

Kent, Matthew

Kerr, Richard

Kess, Tony

Khatib, Hasan

Kielland-Brandt, Morten

Kim, Hyun-Eui

Kim, Jaebum

Kim, John

Kim, TaeSoo

King, Elizabeth

Kirienko, Natalia

Kito, Keiji

Klápště, Jaroslav

Klein, Hannah

Kocher, Thomas

Koelle, Michael

Kohn, Andrea

Kondrashov, Fyodor

Konkin, David

Kowalski, Madeline

Krabbenhoft, Trevor

Krantz, David

Kronstad, James

Krutovsky, Konstantin

Kumar, Satish

Kuo, Chih-Horng

Kupiec, Martin

Kurant, Estee

Kusaba, Makoto

Labbé, Jean-Claude

Labbé, Simon

LaBella, Abigail

Labouesse, Michel

Lacefield, Soni

Lachance, Marc-André

Lado, Bettina

Lai, Eric

Landry, Joseph

Langdon, Quinn

Lara, Leticia

Larkin, John

Larsson, Jan

Lasko, Paul

Lattao, Ramona

Lauer, Edwin

Lazetic, Vladimir

Leakey, Andrew

Lebestky, Tim

LeDeaux, John

Lee, Grace

Lee, Soo

Lee, Teresa

Lee, Tzumin

Leeb, Tosso

Legarra, Andres

Lehermeier, Christina

Lehner, Kevin

Lei, Li

Leips, Jeff

Lenhart, Kari

Levina, Anna

Levy, Daniel

Levy, Julien

Levy, Sasha

Li, Fei

Li, Jinhua

Li, Na

Li, Qingyun

Li, Rongpeng

Li, Sheena

Li, Taihui

Li, Wanlong

Li, Xianran

Li, Xu

Li, Zitong

Liang, Zhikai

Liao, Yi

Lima, Dayane

Lindgren, Gabriella

Lindsey, Amelia

Lipka, Alexander

Liu, Feng

Liu, Hanqiang

Liu, Sanzhen

Ljungdahl, Per

Lloyd, Andrew

Lo, Te-Wen

Lobner-Olesen, Anders

Lohmueller, Kirk

Loidl, Josef

Lok, James

Lonardi, Stefano

Long, Manyuan

Lopez-Cruz, Marco

Loraine, Ann

Lorenz, Aaron

Loreto, Elgion

Lovell, John

Lovett, Susan

Lu, Xiaolei

Ludwig, Adriana

Lupi, Alexa

Lyons, Leslie

Ma, Alan

Macfarlan, Todd

Mack, Katya

MacNeil, Lesley

MacQueen, Amy

Madhani, Hiten

Madlung, Andreas

Mains, Paul

Makunin, Alex

Malkova, Anna

Maloof, Julin

Manhart, Carol

Mansfeld, Ben

Marois, Eric

Martínez, Paulino

Martini, Johannes

Mary-Huard, Tristan

Mata, Juan

Matela, Abigail

Matera, A. Gregory

Matheny, Tyler

Matise, Tara

Mattison, Christopher

Matus, David

Matute, Daniel

Mavian, Carla

McCall, Kimberly

McCoy, Rajiv

McDonald, Jocelyn

McDonald, Megan

McEwan, John

McGaughey, David

McGrath, J

McJunkin, Katherine

McKain, Michael

McKenna, Duane

McLay, Todd

McRae, Allan

McStay, Brian

Measday, Vivien

Megraw, Timothy

Mehlenbacher, Shawn

Meignin, Carine

Merot, Claire

Meslin, Camille

Michaux, Grégoire

Miest, Joanna

Militello, Kevin

Miller, Danny

Miller, Matthew

Miller, Robert

Mirkin, Sergei

Missaoui, Ali

Mitchell, Aaron

Mitchell, Diana

Mitchell, Robert

Miura, Pedro

Modiano, Jaime

Mohr, Stephanie

Mollinari, Marcelo

Montero-Mendieta, Santiago

Moody, Sally

Moore, Jason

Morano, Kevin

Morota, Gota

Morrell, Peter

Morrissey, John

Mortimer, Tatum

Moss, Eric

Mott, Richard

Moura de Sousa, Jorge

Moye-Rowley, Scott

Moyers, Brook

Muehlbauer, Gary

Mukherjee, Neelanjan

Mulhair, Peter

Neiman, Aaron

Neiman, Maurine

Nelson, Thomas

Newton, Irene

Neyhart, Jeff

Nislow, Corey

Niwa, Ryusuke

Nobori, Tatsuya

Nolan, Tony

Norris, Adam

Nuñez-Acuña, Gustavo

Nystul, Todd

O'Huigin, Colm

Ojeda, Dario

Olafson, Pia

Olatoye, Marcus

Olbricht, Gayla

Olesnicky Killian, Eugenia

Olukolu, Bode

Opperman, Charles

Osley, Mary

Ospina-Giraldo, Manuel

Otyama, Paul

Ou, Shujun

Ozerov, Mikhail

Pai, Athma

Palti, Yniv

Palu, Rebecca

Parham, Peter

Parichy, David

Parish, Audrey

Park, Joong-Ki

Parkin, Isobel

Parsons, Jeremy

Patil, Gunvant

Patten, Manus

Patton, Elizabeth

Paull, Robert

Pearce, Stephen

Peiffer, Jason

Pélissié, Benjamin

Pellegrino, Mark

Peñagaricano, Francisco

Peñaloza, Carolina

Pérez-Enciso, Miguel

Pérez-Rodríguez, Paulino

Peris, David

Perlin, Michael

Petes, Thomas

Petrella, Lisa

Pfliegler, Walter

Pfrender, Michael

Picard, Christine

Pickett, F.

Pidoux, Alison

Piepho, Hans-Peter

Pinto, Brendan

Pires, J.

Pividori, Milton

Poland, Jesse

Ponsero, Alise

Pook, Torsten

Pook, Torsten

Pool, John

Port, Fillip

Posnien, Nico

Potter, Christopher

Poupardin, Rodolphe

Powell, Daniel

Powell, Owen

Prendergast, James

Proctor, Robert

Prom, Louis

Promislow, Daniel

Puchta, Holger

Puckett, Emily

Qiu, Yinjie

Quezada, Marianella

Radanović, Aleksandra

Rahi, Praveen

Ramakers, Jip

Rami, Jean-Francois

Rangan, Prashanth

Rashotte, Aaron

Rasmussen, Carolyn

Rast, Jonathan

Rastas, Pasi

Rastas, Pasi

Rau, Andrea

Reddy, Anireddy

Reddy, Umesh

Rellan-Alvarez, Ruben

Rendón, Martha

Reyes-Lamothe, Rodrigo

Reynolds, Richard

Rhind, Nicholas

Rhodes, Davina

Rhodes, Johanna

Rice, Rice

Richards, Christina

Richards, Stephen

Rideout, Elizabeth

Rincent, Renaud

Rine, Jasper

Ritland, Kermit

Robledo, Diego

Rochaix, Jean-David

Rocheleau, Christian

Rödelsperger, Christian

Rodriguez de la Vega, Ricardo

Rodríguez-Medina, José

Rodríguez-Valera, Francisco

Rogers, Rebekah

Rojas, Alejandro

Rokas, Antonis

Ronnegard, Lars

Rousselle, Marjolaine

Runcie, Daniel

Rusche, Laura

Saba, Laura

Salinas, Francisco

Salmon, Armel

Salojärvi, Jarkko

Salvador, Anna

Salz, Helen

Salzberg, Steven

Samaddar, Anirban

Samuk, Kieran

San-Segundo, Pedro

Sanchez-Herrero, Ernesto

Sandhu, Karansher

Santantonio, Nicholas

Santos, Jhonathan

Sardana, Richa

Sasaki, Eriko

Satta, Yoko

Saupe, Sven

Schacherer, Joseph

Schaeffer, Stephen

Schiffer, Philipp

Schlötterer, Christian

Schmidt, Thomas

Schmitz, Lienhard

Schmitz, Robert

Schraiber, Joshua

Schrank, Augusto

Schrauf, Matías

Schrider, Daniel

Schroeder, Nathan

Schwarz, Erich

Schwessinger, Benjamin

Scott, Jeffrey

Scott, Michael

Scully, Erin

Seabury, Christopher

Seidel, Hannah

Seim, Inge

Sekhon, Rajandeep

Selmecki, Anna

Sethupathy, Praveen

Shannon, Laura

Sharbrough, Joel

Sharma, Prashant

Sharma, Santosh

Shechner, David

Shewaramani, Sonal

Shi, Qiong

Shi, Xiaolong

Shi, Xiaowen

Shultz, Allison

Siegele, Deborah

Silva Pereira, Cristina

Silva, Paula

Sim, Sheina

Simecek, Petr

Singh, Jugpreet

Singh, Nadia

Sivasankaran, Sathesh

Skelly, Daniel

Sledziewska-Gojska, Ewa

Slomnicka, Renata

Smith, Chris

Smith, Jeramiah

Snodgrass, Samantha

Sober, Siim

Sobreira, Nara

Sokol, Nicholas

Song, Rentao

Soto, Martha

Sousa, Vitor

Spannagl, Manuel

Spealman, Pieter

Srivastava, Avi

Stahlke, Amanda

Stajich, Jason

Stark, Jeremy

Starr, Daniel

Starz-Gaiano, Michelle

Stavoe, Andrea

Steczkiewicz, Kamil

Steenwyk, Jacob

Stefanini, Irene

Steibel, Juan

Stellwagen, Sarah

Sterken, Mark

Stevison, Laurie

Stirling, Peter

Stolle, Eckart

Straub, Shannon

Street, Nathaniel

Stromvik, Martina

Stupar, Robert

Sturrock, Marc

Sudmant, Peter

Sugino, Ryuichi

Sunnerhagen, Per

Sutani, Takashi

Swanson, Willie

Swarts, Kelly

Szabo, Les

Szpiech, Zachary

Tabima, Javier

Tabuchi, Masashi

Taguchi, Kazunori

Taipale, Mikko

Taliaferro, J. Matthew

Taylor, Julian

Templin, Andrew

Tessema, Biructawit

Thomas, W.

Thompson, Richard

Thorpe, Peter

Thummel, Ryan

Thyme, Summer

Timp, Winston

Titorenko, Vladimir

Tiwari, Vijay

Tobias, Christian

Tobias, Peri

Tollis, Marc

Tolmasky, Marcelo

Tomkiel Dean, John

Tootle, Tina

Toro, Miguel Angel

Torres, Tatiana

Tort, Jose

Townsend, Jeffrey

Tralamazza, Sabina

Tsai, Isheng

Tsuchimatsu, Takashi

Tullet, Jennifer

Turner-Hissong, Sarah

Turner, Caroline

Turner, Jeffrey

Udall, Joshua

Usher, Jane

Valdes, Jorge

Van Kaam, Jan-Thijs

Van Leeuwen, Jolanda

Varadharajan, Srinidhi

Varani, Alessandro

Varney, Rebecca

Vasu, Sheeba

Vaughn, Justin

Vedanayagam, Jeffrey

Verheyen, Esther

Verma, Sujeet

Vincent, Ajoy

Vogl, Claus

Vondras, Amanda

Vonesch, Sibylle

Walters, James

Walworth, Nancy

Wan, Fanghao

Wang, Jinliang

Wang, Laingjiang

Wang, Lihao

Wang, Lixia

Wang, Wen

Wang, Xiaodong

Wang, Xutong

Wang, Zheng

Wang, Zhiyong

Warkentin, Tom

Warringer, Jonas

Wassarman, David

Waterhouse, Robert

Watters, Michael

Watts, Jennifer

Weake, Vikki

Wedekind, Claus

Wei, Kevin

Wei, Lijuan

Wei, Shu-Jun

Weis, Virginia

Weisman, Ronit

Wellband, Kyle

Wellinger, Raymund

Wells, Kristen

Wessells, Robert

West, Alexander

Wheeldon, Ian

Whelan, Fiona

Whibley, Annabel

White-Cooper, Helen

White, Kristin

Wildonger, Jill

Williams, Amy

Wilson, Rod

Wimmers, Klaus

Winderickx, Joris

Wit, Ernst

Wittmeyer, Kameron

Wolfe, Kenneth

Wolters, John

Wong, Alex

Woodruff, Gavin

Wright, Stephen

Xie, Jun

Xie, Shaojun

Xin, Wang

Xu, Jianlong

Xu, Meiying

Xue, Angli

Xue, Chaoyang

Xue, Katherine

Yamamoto, Shinya

Yan, Huihuang

Yandell, Brian

Yang, Feng

Yang, Lei

Yang, Liandong

Yang, Mingyao

Yang, Xiang-Jiao

Yang, Yan

Yang, Yongzhi

Yao, Meng-Chao

Yarden, Oded

Yassin, Amir

York, Daniel

Young, Ry

Yu, Jae-Hyuk

Yu, Jianming

Yusuf, Leeban

Zagoskin, Maxim

Zaman, Luis

Zamanian, Mostafa

Zhai, Jixian

Zhan, Shuai

Zhang, Aimin

Zhang, Dabao

Zhang, Liwu

Zhang, Sheng

Zhao, Lei

Zhao, Yusheng

Zhao, Zhigang

Zheng, Chaozhi

Zhong, Shaobin

Zhou, Wenbin

Zhou, Yongfeng

Zhou, Zhengkui

Zhu, Qian-Hao

Zou, Fei

Zuther, Ellen

